# Postoperative anemia is a risk factor for acute kidney injury after open aorta and vena cava surgeries

**DOI:** 10.1371/journal.pone.0240243

**Published:** 2020-10-13

**Authors:** Rui Cui, Fangda Li, Jiang Shao, Yuzhu Wang, Cai Yue, Yuehong Zheng, Xuemei Li

**Affiliations:** 1 Department of Nephrology, Peking Union Medical College Hospital, Chinese Academy of Medical Sciences, Beijing, China; 2 Department of Nephrology, Beijing Haidian Hospital and Beijing Haidian section of Peking University Third Hospital, Beijing, China; 3 Department of Vascular Surgery, Peking Union Medical College Hospital, Chinese Academy of Medical Sciences, Beijing, China; Cleveland Clinic, UNITED STATES

## Abstract

Open aorta and vena cava surgeries are usually associated with substantial blood loss which may result in postoperative acute kidney injury (AKI). The present study is designed to investigate the prevalence, outcome and risk factors of postoperative AKI associated with open aorta and vena cava surgeries, with a focus on the role of anemia in these conditions. A retrospective review of medical records of Peking Union Medical College Hospital was conducted. Patients who underwent open aorta and vena cava surgeries during January 1, 2010 and June 30, 2014 were included in this study. The primary analysis was between patients underwent open aorta and vena cava surgeryies, with or without postoperative AKI. Multivariable logistic regression models were used to determine risk factors of postoperative AKI. The study included 79 patients (63.3% male) with a mean age of 52.5±17.3 years (range, 17–81 years). Postoperative AKI occurred in 23/79 (29.1%) of the patients. Anemia was present in 11/79 (16%) at baseline, and increased to 45/79 (52%) postoperatively. After adjustment for various risk factors, postoperative anemia (OR, 5.202; 95% CI 1.403–19.285) was independently associated with postoperative AKI. AKI is a common complication in patients who undergo open aorta and vena cava surgeries, and postoperative anemia was the most relevant predictive factor of AKI. Strategies to minimize bleeding and anemia for all patients may be advisable. Further studies are needed to assess the impact of AKI on long term outcome and to examine preventive strategies to address potentially modifiable risk factors.

## Introduction

Acute kidney injury (AKI) is a prevalent and prognostically significant complication in surgical patients. Depending on the AKI definitions adopted and the population studied, AKI occurs in up to 16% - 48% of surgical patients [1–3]. AKI after any type of surgery is independently associated with substantially worse outcomes, including higher short-term mortality, prolonged length of hospital stay, worsened long-term survival, and increased risk of end stage renal disease (ESRD) [4–8].

Despite advances of endograft techniques, open aorta and vena cava surgeries are still used in patients unsuitable for endovascular repair, and are associated with substantial incidence of postoperative AKI [9, 10]. These procedures are usually associated with significant blood loss, which may result in hypovolemia or hypoxemia. Regionally reduced oxygen supply lead to physiologically low oxygen tensions within the renal cortex and the medulla and may cause postoperative AKI [2, 3]. However, most previous researches focused on AKI after endovascular procedures which may be associated with contrast use, and the prevalence, risk factors and outcomes of AKI after open aorta and vena cava surgeries were poorly addressed in the literatures. Hence we performed an analysis in patients underwent open surgeries of major vasculatures including aorta and vena cava, to investigate the prevalence, outcome and risk factors of postoperative AKI, with a focus on the role of anemia in these conditions.

## Materials and methods

This study was approved by the Institutional Review Board (IRB) of Peking Union Medical College Hospital the University (S-K048).

### Database and participants

We conducted a retrospective review of the medical records of patients during September to November, 2015. The patients underwent vascular surgeries at Peking Union Medical College Hospital from January 1, 2010 to June 30, 2014. The medical records were accessed on patients who underwent vascular surgeries of all types, and cases involving open aorta and vena cava surgery were included in this study. Cases that involved contrast administration during the perioperative period, perioperative death, or with incomplete data were excluded from the analysis. Patient demographics and perioperative data were obtained from the chart review. Informed consents were obtained by the time of patient admission for later access of their medical records. Authors had access to information that could identify individual participants during or after data collection.

### Covariates and definitions

The primary analysis was between patients underwent open surgeries of aorta or vena cava, with or without postoperative AKI, to evaluate the risk factors associated with postoperative AKI. AKI was defined as ≥26.5 μmol/L increase in serum creatine (SCr) level within the first 48 hours post-operation, or an increase in SCr levels from baseline by more than 50% during the first 7 postoperative days, according to the AKI criteria from the Kidney Disease: Improving Global Outcomes (KDIGO) Clinical Practice Guideline for Acute Kidney Injury, 2012 [11]. AKI was staged for severity according to the criteria presented in Table 1. Anemia: hemoglobin (Hb) (male) <120g / l, Hb (fmale) <110g / l. (Other definitions are included in Tables 2 and 3). Chronic kidney diseases (CKD): GFR<60ml/min/1.73m^2^. Hypoproteinemia: albumin <30g / l. Coronary heart disease: left ventricular ejection fraction (LVEF) <40%. Blood loss: anesthesiologist estimated with blood recovered intraoperative blood salvage. Using of vasopressors: using the vasopressors drugs during operation.

**Table 1:** Staging of acute kidney injury.

Stage	Serum creatinine
1	1.5 to 1.9 times baseline or≥0.3 mg/dl (≥26.5 μmol/l) increase
2	2.0 to 2.9 times baseline
3	3 times baseline or increase to ≥353.6 μmol/L in SCr or initiation of renal replacement therapy

**Table 2:** Patients demographics and characteristics.

	*All (N = 79)*	*AKI (N = 23)*	*No AKI (N = 56)*	*P*
	Mean (± SD) or median (IQR) or *N* (%)	Mean (± SD) or median (IQR) or *N* (%)	Mean (± SD) or median (IQR) or *N* (%)	
**Demographics**				
Age, years	52.5(±17.3)	55.8(±15.2)	51.2(±18)	0.25
Male sex	50(63.2%)	14(60.9%)	36(64.3%)	0.82
BMI, kg/m^2^	23.04±2.45	23.18±2.24	22.98±2.54	0.32
Ace inhibitors/ ARBs use	39(49.4%)	14(60.9%)	25(44.6%)	0.20
**Comorbidities**				
Hypertension	38(48.1%)	14(60.9%)	24(42.9%)	0.15
Coronary heart disease	6(7.5%)	2(8.7%)	4(7.1%)	0.813
Hypoproteinemia ^a^	9(14.3%)	4(25%)	5(10.6%)	0.315
CKD	9(11.4%)	3(13.0%)	6(10.7%)	0.12
Diabetes	2(2.5%)			
Baseline Scr, μmol/l	74.0(±24.8)	75.4(±27.2)	73.5(±23.9)	0.758
Peak Scr, μmol/l	95.1(±38.0)	124.2(±47.6)	83.2(±25.0)	0.001
**Surgery types**				
Renal artery aneurysm repair	8(10.1%)	6(26.1%)	2(3.6%)	0.009
Inferior vena cava leiomyosarcoma resection	6(7.6%)	3(13.0%)	3(5.4%)	0.48
Aortic aneurysm repair	31(39.2%)	8(34.8%)	23(41.1%)	0.6
Aortic bypass	16(20.3%)	3(13.0%)	13(23.2%)	0.48
Other surgeries	18(22.8%)	3(13.0%)	15(26.8%)	0.3
**Intra-operative conditions and management**				
Baseline MAP, mmHg	99.0(±13.6)	105(±14)	97(±13)	0.017
Nadir of Intraoperative MAP, mmHg	71.5(±9.5)	72(±10)	71(±9)	0.963
Vasopressors use	12(15.2%)	5(21.7%)	7(12.5%)	0.282
Antibiotics use	61(77.2%)	19(82.6%)	42(75%)	0.47
Fluid infusion, ml	4314(3000,5100)	4600(3700,6500)	4150(3000,4775)	0.094
Suprarenal aortic cross clamp	11(13.9%)	5(21.7%)	6(10.7%)	0.316
Surgical duration, min	420.76±117.49	469.13±142.475	400.89±100.39	0.018

*P* value denotes comparison to AKI and non-AKI subgroups; *AKI*, acute kidney injury; *CKD*, chronic kidney diseases; *SCr*, serum creatinine; SD: standard deviation, IQR: interquartile range

^a^The data is not complete.

**Table 3:** Perioperative anemia of patients with and without acute kidney injury (AKI).

	*All (N = 79)*	*AKI (N = 23)*	*No AKI (N = 56)*	*P*
	Mean (± SD) or median (IQR) or *N* (%)	Mean (± SD) or median (IQR) or *N* (%)	Mean (± SD) or median (IQR) or *N* (%)	
Blood loss during operation, ml	1000(700,2000)	1200(800,2500)	1000(600,1950)	0.042
Transfusion	41(56.9%)	15(69.6%)	26(46.4%)	0.162
Baseline Hb, g/l	130.7(±20.2)	124.9(±20.5)	132.8(±19.9)	0.14
Postoperative Hb, g/l	112.4(±19.5)	104.2(±16.3)	115.4(±19.9)	0.02
Preoperative anemia	11(13.9%)	4(21.1%)	7(13.0%)	0.4
Postoperative anemia	46(58.2%)	17(74.0%)	29(51.8%)	0.049

*Hb*: hemoglobin.

SD: standard deviation, IQR: interquartile range.

The baseline renal function was the last serum creatinine before surgery; the peak serum creatinine was the highest value of serum creatinine obtained during the first 7 days after surgery. The baseline levels of hemoglobin (Hb), hematocrit (HCT) and albumin (Alb) were obtained from the latest preoperative data, and the first test results after surgery were used as the postoperative results.

### Data analyses

Data were expressed as a mean ± SD for continuous variables and as percentages for discrete variables. Continuous data were analyzed by Student’s t test for equal variance or the Mann-Whitney test for unequal variance, and categorical valuables were investigated by the Pearson χ^2^ or Fisher’s exact test. A two-sided *P* value < .05 was considered significant. Logistic regression (adjusted for multiple covariates) was used to determine the odds ratio (OR) and the 95% confidence intervals (CI) associated with and without AKI. To adjust for additional unmeasured confounding variables, we fit a logistic regression model using the aforementioned covariates with the procedure type as the dependent variable. All covariates with *P* < .3 from univariate analysis were put into logistic regression model. Statistical analyses were performed using SPSS (Version 20.0. for Windows; SPSS, Inc.), and significance was assigned at *P* values < .05.

## Results

### Clinical characteristics

A review of the medical records revealed 4926 cases of vascular surgery in Peking Union Medical College Hospital from January 1, 2010 to June 30, 2014. A total of 79 cases met the inclusion criteria, accounting for 1.6% of the population. The clinical features of the 79 cases are summarized in Table 2. The patient population included 50 males and 29 females, with a male predominance (63.3%). The mean age at the time of surgery was 52.5±17.3 years (range, 17–81 years). Among the patients, 48.1% (38) had a medical history of hypertension, and the prevalence of CKD, coronary heart disease and diabetes were 11.4% (9), 7.5% (6), and 2.5% (2), respectively. 31 patients (39.2%) underwent open aortic aneurysm repair, 16 patients (20.3%) underwent aortic bypass, 8 patients (10.1%) underwent renal artery aneurysm repair, 6 patients (7.6%) underwent inferior vena cava leiomyosarcoma resection, and 18 patients (22.8%) underwent other major vascular surgeries, including superior mesenteric artery aneurysm repair, splenic aneurysm repair, and common iliac artery aneurysm repair. AKI occurred in 23 (29.1%) of the patients, with stage 1 AKI in 19 patients (82.6%), stage 2 in 3 patients (13.0%), and Stage 3 in 1 patient (4.3%). The demographic characteristics and intraoperative data for the study participants are shown in Tables 2 and 3. No significant differences were observed between the 2 groups in age, sex, preoperative blood pressure, BMI, Ace inhibitors/ARBs use and preexisting comorbidities such as hypertension, hypoalbuminemia and CKD. The mean baseline serum creatinine in both the AKI and non-AKI groups was similar.

### Perioperative characteristics of patients with AKI

**Fig 1:**
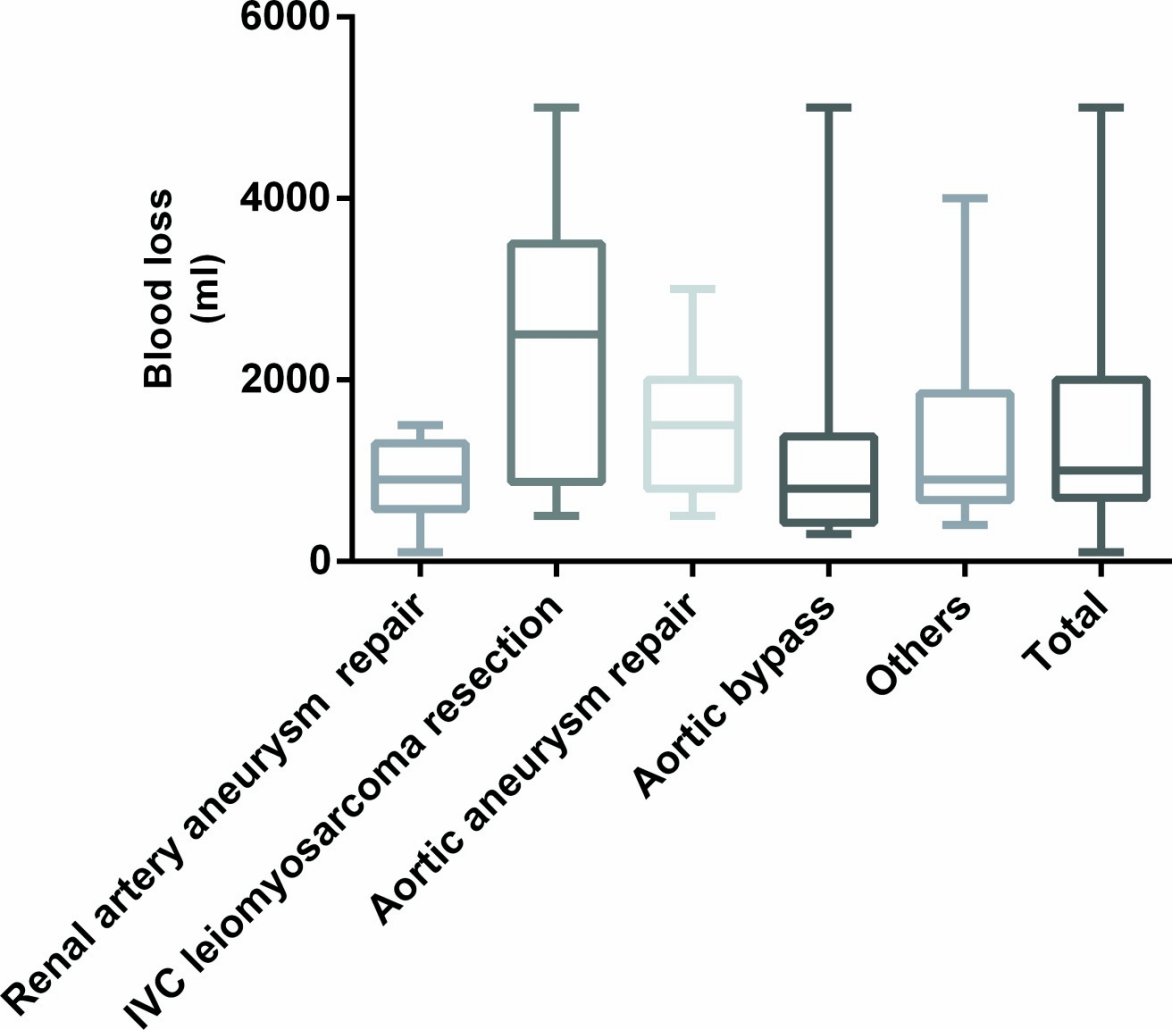
Blood loss of different surgery types. IVC, Inferior vena cava.

**Fig 2:**
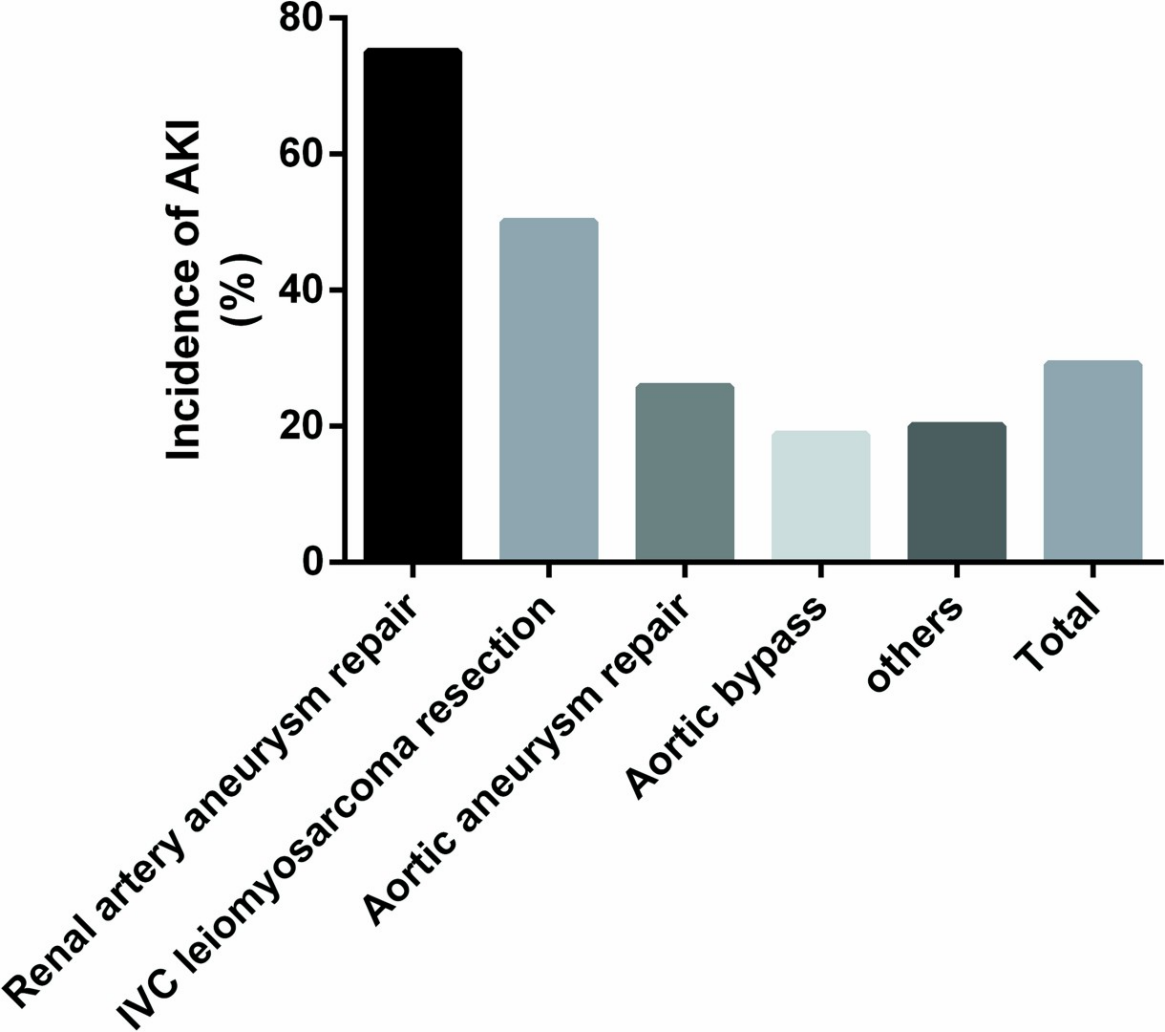
Incidence of post-operative acute kidney injury of different surgery types. IVC, Inferior vena cava.

Among all of the cases studied, the surgical duration of AKI groups was longer than the non-AKI groups (469.13±142.475min vs 400.89±100.39 min, *p* = .018). The median blood loss (ml) during the operation was 1000 (700, 2000). The mean of intraoperative transfusion of packed red blood cells was 1.99 ± 2.86 units and intraoperative transfusion of plasma was 255.70 ± 430.78 ml A total of 11/79(16%) of the patients had preoperative anemia; postoperatively, the occurrence of anemia increased to 45/79(52%). There was a significant difference in intraoperative blood loss (ml) between the AKI (1200 [800, 2500]) and the non-AKI (1000 [600,1950]) patients. Transfusion rates in AKI and non-AKI was 65.2% (15/23) and 46.4% (26/56) respectively. In particular, the incidence of AKI increased in patients with postoperative anemia comparing to those without anemia (37.0% [17/46] vs 18.2% [6/33]). The proportion of patients that received vasopressors during operations were 21.7% (5/23) and 10.7% (7/56) among the AKI and non-AKI patients, respectively. The time of suprarenal aortic cross clamp was 41.67±22.39min. Use of suprarenal aortic cross clamp during the operation was associated with an increased incidence of AKI (45.5% [5/11] vs 26.5% [18/68]). The mean blood loss of the patients who received vasopressors was 1764 ml vs 1361 ml in those who did not. The mean blood loss in patients underwent renal artery aneurysm repair was 900±160 ml. The maximum amount transfusion of packed red blood cells was 2 units and the mean was 0.25±0.7 units. The maximum amount transfusion of plasma was 400ml and the mean was 50±141ml. 2 units and the mean was 0.25±0.7 units. The maximum amount of intraoperative salvage autotransfusion was 1907ml and the mean was 502.5±632.6ml. and 75.0% of these patients developed AKI. The mean blood loss of patients who underwent inferior vena cava leiomyosarcoma resection was 2400±650 ml. The maximum amount transfusion of packed red blood cells was 13 units and the mean was 7.17±4.83 units. The maximum amount transfusion of plasma was 1000ml and the mean was 533.33±467.62ml. The maximum amount of intraoperative salvage autotransfusion was 1037ml and the mean was 322.83±502ml. and 50% of these patients developed AKI. The mean blood loss in patients who underwent open aortic aneurysm repair was 1500±160 ml. The maximum amount transfusion of packed red blood cells was 10 units and the mean was 1.94±2.54 units. The maximum amount transfusion of plasma was 2000ml and the mean was 219.35±439.26ml. The maximum amount of intraoperative salvage autotransfusion was 2300ml and the mean was 559.43±480.69ml. and 25.8% of these patients developed AKI. The incidence of AKI among patients underwent aortic bypass surgery was 18.8%, and the incidence in patients underwent other major vascular surgeries was 20% (Figs 1 and 2).

### Risk factor of AKI

Open aorta and vena cava surgery, postoperative anemia, renal artery aneurysm repair, blood loss, and the use of intraoperative vasopressors, were all associated with AKI on univariate analysis (Tables 2 and 3). In the multivariate analysis, postoperative anemia (OR, 5.202; 95% CI, 1.403~19.285) was an independent predictor of postoperative AKI, but not age (OR, 1.015; 95% CI, 0.981~1.049), hypertension (OR, 1.586; 95% CI, 0.511~4.919), CKD (OR, 0.126; 95% CI, 0.011~1.391), or vasopressor use (OR, 4.577; 95% CI, 0.988~21.194) (Table 4).

**Table 4:** Risk factors of postoperative acute kidney injury.

	*P*	*OR*	95% *CI*
Age, years	0.396	1.015	0.981–1.049
Hypertension	0.425	1.586	0.511–4.919
CKD	0.091	0.126	0.011–1.391
Vasopressors use	0.052	4.577	0.988–21.194
Postoperative Anemia	0.014	5.202	1.403–19.285

*CI*, confidence interval; *OR*, odds ratio; *CKD*, chronic kidney diseases.

## Discussion

Despite the development of endovascular procedures, open surgeries of aorta and vena cava are still used, especially in patients who are not suitable for endovascular procedures, and are associated with substantial mortality and complicated courses [1–3]. AKI in a common complication associated with open surgeries of aorta and vena cava. However, the incidence and risk factors of AKI in these conditions have not been adequately addressed.

Ischemic-reperfusion injury is common following surgeries and causes AKI [12–14]. The incidence of AKI after surgery was different in different studies, and it was mainly related to the observation from different patients [3, 15]. In our study, we assessed the incidence and risk factors of AKI in patients underwent open surgery of aorta and vena cava. The prevalence of postoperative AKI was as high as 29.1%, indicating that AKI is a common complication in these patients. The patients included in this study underwent open surgery of aorta and vena cava, which may be extremely difficult due to the complicated anatomic structures and abundant collateral circulations [9, 16]. These procedures are usually accompanied with prolonged length of surgery, higher bleeding risk, and more complicated perioperative course, placing the patients at increased risk of AKI [3, 8, 17]. In our study, the surgical duration of postoperative AKI groups was longer than that of no-AKI groups. The importance of AKI as a risk factor for mortality and we need to focus on high-risk patients for prevention and early identification of AKI and prompt treatment. However, we adopted the 2012 KIDIGO definition of AKI in our study, which is still based on increases in serum creatinine concentrations. New biomarkers of kidney injury, such as serum cystatin C (ScysC) Neutrophil Gelatinase-Associated Lipocalin (NGAL) and brain natriuretic peptide (BNP) may facilitate earlier detection of renal injury, and thus early intervention aimed at limiting the associated morbidity and mortality [18–20].

Postoperative AKI has been shown to be associated with early and long-term mortality and long-term risk of ESRD [4, 5, 21]. Thus adequate attentions are needed pertaining AKI following these surgeries.

It is noteworthy that AKI is more prevalent among those patients with more blood loss in our study. The mean blood loss during leiomyosarcoma and aortic aneurysm surgeries were 2400 ± 650 ml and 1500 ± 160 ml, and incidences of AKI after these procedures were up to 50% and 25.8%, respectively. Bleeding and postoperative anemia were associated with postoperative AKI, and multivariate analysis showed that postoperative anemia was an independent risk factor for AKI (OR, 5.202; 95% CI, 1.403–19.285). And vasopressors use during surgeries, which probably indicate more blood loss during the procedures, is associated with higher risk of postoperative AKI.

However, even without massive hemorrhage, the incidence of AKI after open renal artery aneurysm repair was higher comparing to other surgical procedures of the aorta and vena cava. Aortic cross clamping was a renal hazard for this group of patients, and AKI occurred in 21.5% of patients who underwent surgery with a suprarenal aortic cross clamp compared to 12.5% in those who didn’t. Aortic cross clamping blocks effective renal perfusion and exposes the renal parenchyma to reduced oxygen tension, contributing to ischemia/reperfusion injury [14].

The kidney is especially vulnerable to hypoxic injury. Acute blood loss, hypovolemia, hemodilution or renal artery blocking during surgical processes may reduce regional oxygen supply, leading to physiological low oxygen tensions within the renal cortex and the medulla and microvascular dysfunction, and may result in acute kidney injury [12–14]. Furthermore, these conditions are associated with increased renal tubular oxygen consumption and oxidative stress, further confounding the imbalance of oxygen supply and demandz [13, 14].

Postoperative anemia is the main predictor of postoperative AKI in our study, and other baseline characteristics that have been traditionally associated with AKI after other surgery types including age, baseline renal function, hypertension, and diabetes were not associated with postoperative AKI in our study. This may be attributed to the patient population selected, and limited case number in our study. Larger, multicenter, prospective studies may be needed to verify these conclusions.

Therefore, among patients undergoing open vascular surgery of aorta or vena cava, prevention and treatment may include avoiding unnecessary blood loss, ensuring optimal renal perfusion and ameliorating postoperative anemia. However, as shown in previous studies, treating perioperative anemia with blood transfusion didn’t improve renal outcomes of patients undergoing surgery [22–24]. Furthermore, receiving intraoperative blood transfusion was associated with a higher risk of postoperative mortality, pulmonary complications, renal failure and infectious complications. Transfusions were associated with cytokine release, bacterial contamination, sepsis, metabolic disturbances, circulatory overload, hemolytic reactions, and risk of old blood [25]. Given these findings, other means to alleviate perioperative anemia are needed to consider. Erythropoietin (EPO) is a natural hormone produced in human body, and is a regulator of erythroid precursor cells. Evidences suggest that EPO has anti-apoptotic, anti-oxidative and anti-inflammatory properties, and is able to reduce the injury caused by ischemia/reperfusion of kidney in animal model [26, 27]. Thus, EPO might be considered as a possible prophylactic intervention to prevent postoperative AKI.

This study has several limitations that need to be considered. First, the effects of unmeasured confounders on these relationships we describe cannot be dismissed because this is an observational study. Potentially important unmeasured confounders include the use of perioperative medications that may influence kidney function. Second, this is a single-center study with limited cases, and larger, multicenter, and prospective studies may be needed to further validate our finding.

## Conclusions

In our study, AKI is a common complication in patients who undergo open aorta and vena cava surgeries, and postoperative anemia is the most relevant predictive factor of AKI. Strategies to minimize bleeding and anemia for all patients may be advisable. Further studies are needed to assess the impact of AKI on long term outcome and to examine preventive strategies to address potentially modifiable risk factors.

## Supporting information

S1 File (SAV)
